# Miscanthus Establishment and Overwintering in the Midwest USA: A Regional Modeling Study of Crop Residue Management on Critical Minimum Soil Temperatures

**DOI:** 10.1371/journal.pone.0068847

**Published:** 2013-07-03

**Authors:** Christopher J. Kucharik, Andy VanLoocke, John D. Lenters, Melissa M. Motew

**Affiliations:** 1 Department of Agronomy, University of Wisconsin-Madison, Madison, Wisconsin, United States of America; 2 Nelson Institute Center for Sustainability and the Global Environment, University of Wisconsin-Madison, Madison, Wisconsin, United States of America; 3 Department of Energy Great Lakes Bioenergy Research Center, University of Wisconsin-Madison, Madison, Wisconsin, United States of America; 4 Department of Atmospheric Sciences, University of Illinois, Urbana, Illinois, United States of America; 5 School of Natural Resources, University of Nebraska-Lincoln, Lincoln, Nebraska, United States of America; Virginia Commonwealth University, United States of America

## Abstract

Miscanthus is an intriguing cellulosic bioenergy feedstock because its aboveground productivity is high for low amounts of agrochemical inputs, but soil temperatures below −3.5°C could threaten successful cultivation in temperate regions. We used a combination of observed soil temperatures and the Agro-IBIS model to investigate how strategic residue management could reduce the risk of rhizome threatening soil temperatures. This objective was addressed using a historical (1978–2007) reconstruction of extreme minimum 10 cm soil temperatures experienced across the Midwest US and model sensitivity studies that quantified the impact of crop residue on soil temperatures. At observation sites and for simulations that had bare soil, two critical soil temperature thresholds (50% rhizome winterkill at −3.5°C and −6.0°C for different Miscanthus genotypes) were reached at rhizome planting depth (10 cm) over large geographic areas. The coldest average annual extreme 10 cm soil temperatures were between −8°C to −11°C across North Dakota, South Dakota, and Minnesota. Large portions of the region experienced 10 cm soil temperatures below −3.5°C in 75% or greater for all years, and portions of North and South Dakota, Minnesota, and Wisconsin experienced soil temperatures below −6.0°C in 50–60% of all years. For simulated management options that established varied thicknesses (1–5 cm) of miscanthus straw following harvest, extreme minimum soil temperatures increased by 2.5°C to 6°C compared to bare soil, with the greatest warming associated with thicker residue layers. While the likelihood of 10 cm soil temperatures reaching −3.5°C was greatly reduced with 2–5 cm of surface residue, portions of the Dakotas, Nebraska, Minnesota, and Wisconsin still experienced temperatures colder than −3.5°C in 50–80% of all years. Nonetheless, strategic residue management could help increase the likelihood of overwintering of miscanthus rhizomes in the first few years after establishment, although low productivity and biomass availability during these early stages could hamper such efforts.

## Introduction

The recent push towards developing new bioenergy cropping systems has focused on identifying highly productive plants – other than *Zea mays* (maize) – to provide biomass for lignocellulosic bio-refineries [1]. Ideal bioenergy cropping systems should lead to improved soil, water, and air quality across agricultural regions, as well as reducing emissions of greenhouse gases without competing with food sources [2]. One of the plants of interest, miscanthus, is a highly productive C_4_ perennial rhizomatous grass, which is not native to many temperate regions, but its bioenergy potential is now being studied extensively in Europe, the US, and Canada [3]–[5]. Specifically in the Midwest US, *Miscanthus × giganteus* is being studied as a model cellulosic feedstock because for low amounts of agrochemical inputs, its productivity is extremely high, ∼60% higher than maize total aboveground biomass [5], and double that of another C_4_ grass contender, switchgrass (*Panicum virgatum*), regardless of climate and nitrogen fertilizer applied [6].

Before cellulosic feedstocks can supplant maize grain as the dominant source there are significant technical obstacles to overcome [7]. Key barriers include developing an economically viable process to break down cellulose and establishing highly productive plants in environmental conditions that are more harsh than their native regions [8]. In the case of miscanthus, cultivation in the US has largely focused on *Miscanthus* × *giganteus*, which is a natural sterile triploid hybrid of *Miscanthus* × *sinensis* and *Miscanthus* × *sacchariflorus* [9]. Because the triploid *M. × giganteus* clones are sterile, establishment results from the planting of rhizomes at a typical depth of 5 to 10 cm [10], and is therefore more costly to establish on the basis of time and money [11].

A key factor that may affect the viability of rhizome propagation for establishing miscanthus in non-native regions is the susceptibility to damage at cold temperatures [11]. Temperatures below freezing can lead to significant miscanthus production losses in two forms. First, soil temperatures that fall below a critical threshold can damage rhizomes, where vulnerability appears elevated in the first winter after establishment [3]. Second, air temperatures below zero after the emergence of new leaves can damage young vegetation, and rhizomes may not sprout again [1], [3]. There appears to be a wide variation in frost tolerance among different genotypes because miscanthus has been found to exist naturally from warm subtropical regions to more northern locations of the subarctic [12], [13]. Previous research conducted as part of the European Union (EU) Miscanthus Productivity Network [3], [14], [15] suggested that at some sites in northern Europe including Denmark, Ireland, and Germany, rhizomes in newly established stands did not survive the first year (winter). This has led to subsequent research on the cold tolerance of different genotypes, including *M. sacchariflorus* and *M. sinesnsis* [13]. Results suggested that the lethal temperature at which 50% of shoots were killed was −8°C for *M. giganteus*, −7.5°C for *M. sacchariflorus*, and between −6 and −9°C for two hybrids of *M. sinensis* [1], [16]. For rhizomes, the lethal temperature at which 50% of rhizomes were killed was −3.5°C for *M. giganteus* and *M. sacchariflorus*, and −4.5°C and −6°C, respectively for two different hybrids of *M. sinensis* [1], [13].

In the US, research has focused predominantly on *M.* × *giganteus* in the Midwest, a region that typically sees extended periods of cold Arctic air outbreaks during the late fall and winter, and correspondingly is at risk to experience near-surface soil temperatures below 0°C as well as frequent freeze-thaw cycles. These cold temperature dynamics create a wide range of uncertainty concerning overwintering of miscanthus in the Midwest US [7]. Recent research has documented the impacts of rhizome size, planting depth, and cold storage on the success of establishment, and arrived at the conclusion that *M.* × *giganteus* rhizomes are best suited to be planted at a depth of 10 cm [8]. Heaton et al. [7] suggested that at this 10 cm depth in Illinois, mature stands of *M.* × *giganteus* have been able to consistently survive winter air temperatures as cold as −20°C and soil temperatures below −6°C. At the University of Wisconsin-Madison's agricultural research station near Arlington, Wisconsin, multiple plots of *M.* × *giganteus* that originated from hand planted rhizomes in spring of 2008 experienced 90% winterkill during the winter of 2008–09, which was representative of other plantings in the Midwest US that year [7].

Crop residue, the unharvested portion of above-ground biomass, can have a significant impact on the surface energy balance and soil properties including temperature. It has been well documented that surface residue management in agricultural systems can aide in conserving soil moisture by reducing maximum soil temperatures by up to 10°C and decreasing evapotranspiration due to a higher surface albedo [17]–[21]. Residue can also act as an effective insulating barrier to the mineral soil during winter, increasing soil temperatures by 5 to 8°C in the central US [21], [22]. Therefore, improved or adapted agronomic management of *M. × giganteus* residue in the first several years may be key to increasing the likelihood of successful establishment.

Our primary objective was to investigate how the risk of *M. × giganteus* rhizome threatening temperatures could be reduced with strategic residue management. This objective was based on using observations and an agroecosystem model to examine historical spatial and temporal patterns of extreme minimum 10 cm soil temperatures across the Midwest US. Given previous research results, we focus on rhizome losses because this type of damage appears to be more devastating because frost damage to leaves appears to be survivable. Specifically, we created a simulated reconstruction of daily wintertime soil temperatures at high spatial resolution (0.08333°, 5 minute, or ∼10 km) across the Midwest US (a region bounded by 36°N to 50°N lat and −79°W and −105°W lon) from 1948–2007, and quantified the frequency that lethal soil temperature thresholds, previously suggested for two miscanthus genotypes (−3.5°C and −6.0°C), were reached at 10 cm depth. Through model sensitivity studies, we investigate how varied thicknesses of prostrate layers of miscanthus straw and corn residue impact wintertime soil temperatures, and how management post harvest could reduce the risk of miscanthus establishment failure. We hypothesize that a prostrate thatch layer of miscanthus straw or corn residue of 1 cm or greater will increase wintertime soil temperatures compared to removal of the residue post harvest, and reduce the risk of soil temperatures at 10 cm reaching critical rhizome kill thresholds. To address our objectives, we employ a dynamic agroecosystem model, Agro-IBIS [21], [23] that has been recently modified to include representation of miscanthus and switchgrass [24], [25]. We conduct a validation of Agro-IBIS simulated snow depth and 10 cm soil temperatures using several Midwest observational datasets. We conclude with an analysis of trends in the coldest annual soil temperatures to determine whether climate change has led to decreased risk of winterkill of *M. × giganteus*.

## Methods

### Model description

Agro-IBIS is a process-based ecosystem model capable of simulating managed and natural ecosystem dynamics of North America, with coupled carbon, water, and energy cycles. Agro-IBIS was developed by adapting a Global Dynamic Vegetation Model (DGVM), called the Integrated Biosphere Simulator (IBIS) [26], [27], to simulate corn, soybean, and wheat cropping systems across the continental US [28], and most recently miscanthus and switchgrass [24], [25]. Agro-IBIS simulates the energy, water, carbon, and momentum balance of the soil-plant-atmosphere system at a 60-min time step. The model includes two vegetation layers with eight potential forest plant functional types (PFTs) in the upper canopy, and two grasses (cool and warm season) and two shrub PFTs in the lower canopy. Row crops and miscanthus are simulated as part of the lower canopy layer. The model version used in this study includes 11 soil layers of varying thicknesses to a 250 cm depth, which are parameterized with one of eleven soil textural categories and corresponding physical attributes [29]. A three-layer thermodynamic snow model simulates the energy balance of the snow surface and changes in snow cover in terms of temperature, fractional coverage, and total snow thickness [30]. Physiologically-based formulations of leaf-level photosynthesis, stomatal conductance [31]–[33] and respiration control canopy exchange processes, and parameters vary according to generalized vegetation categories (e.g., trees, shrubs, C_3_ and C_4_ grasses or crops). The reader is referred to Li et al. [34] for more details about root water uptake and hydrology and Soylu et al. [35] for description of one dimensional water movement through the soil profile.

Agro-IBIS simulates crop growth transitions through phenological stages of development using an accumulated thermal time approach, and characterizes seasonal changes in carbon (C) allocation to specific crop C pools (i.e. leaf, stem, root, and grain). Leaf area index (LAI) is calculated each timestep using the accumulated leaf tissue C multiplied by a crop specific leaf area value. Canopy and land surface processes in Agro-IBIS are based on the key differences in C_3_ and C_4_ physiology, daily phenology, and carbon allocation so that coupled carbon-water exchange is responsive to agricultural management and environmental stresses. For a complete description of the modeling approach, the reader is referred to several other publications [23], [27], [28], [36]. IBIS and Agro-IBIS have been validated extensively from the individual farm scale to the global scale to improve model formulations and parameterizations [21], [23]–[25], [27], [28], [30], [37]–[41].

### Other modeling approaches and the selection of Agro-IBIS

We briefly review two other well validated models (HYDRUS and SHAW) that are often used to study the impacts of agricultural management on soil heat and water flow and offer reasoning why Agro-IBIS was selected to carry out the study objectives. HYDRUS is a variably saturated soil water flow model that numerically solves the mixed-based Richards' equation for saturated - unsaturated water flow and heat transport [42], [43]. HYDRUS simulates water and heat movement in one-dimensional, variably saturated homogeneous media and represents infiltration, evaporation, root water uptake and transpiration, soil water storage, deep drainage, and groundwater recharge. The Simultaneous Heat And Water (SHAW) model is a one-dimensional physically based model of water and heat transport in soils [44], and is capable of simulating infiltration, evapotranspiration (ET), interception, and other hydrologic processes. SHAW has been used to address soil tillage and residue effects on soil freezing and soil water conservation [45] and the interaction between vegetation, soil properties, and other land surface characteristics on frozen soil processes [46]. The Agro-IBIS soil physics module uses Richard's equation to calculate the time rate of change of liquid soil moisture, and the vertical flux of water is modeled according to Darcy's Law. The water budget of soil is controlled by the rate of infiltration, evaporation of water from the soil surface, the transpiration stream originating from plants, and redistribution of water in the profile [27]. Each soil layer in Agro-IBIS is described in terms of soil temperature, volumetric water content, and ice content for any time step [27].

Therefore, while the three models discussed have similar capabilities in simulating soil heat and water flow, HYDRUS and SHAW have some limitations that make them difficult to apply to this study. First, they are designed as point models, and therefore operate at a limited spatial scale compared to Agro-IBIS, which makes it difficult to answer questions across broad regional scales. Second, HYDRUS and SHAW require temporal changes in leaf area index (LAI) and some other vegetation characteristics to be input by the user; therefore, they cannot explicitly simulate phenological development, growth (i.e. photosynthesis), or differences in carbon allocation among different plant species such as miscanthus and corn. Lastly, HYDRUS and SHAW do not explicitly represent carbon and nutrient cycling coupled to water and energy exchanges in the soil-plant-atmosphere system. Therefore, in the context of studying the impact of variable plant growth and development, litter decomposition, and residue management from 1978–2007 on the water cycle and the magnitude of minimum wintertime soil temperatures, HYDRUS and SHAW have limited abilities to generate a historical record for the entire Midwest USA. While SHAW and HYDRUS are exceptional modeling tools in their own right, the expanded capabilities of Agro-IBIS made it a better choice for this particular study.

### Agro-IBIS inputs

Climate inputs required at each model time step for each grid cell include solar radiation, temperature, precipitation, relative humidity, and wind speed. ZedX Inc. (Bellefonte, PA) developed a daily gridded weather dataset at 10 km resolution (0.08333°) for a sixty-year period from 1948 through 2007, which included all six variables needed as model input. ZedX Inc. (Bellefonte, PA) generated the gridded weather data using statistical interpolation of observational data that was subject to a rigorous quality control procedure. Before the data were input into the interpolation algorithm, quality control checks were performed which included assessments of plausibility, checks against observational extremes, and checks against neighboring stations using a quality control threshold based on standard deviations. The spatial extent of this dataset spanned from 24°N to 52°N latitude, and 50°W to 130°W longitude. Three data sets were used to generate the gridded maximum and minimum daily temperatures and precipitation. Input station data for Canada and Mexico were obtained from the Global Historical Daily Climatology (GHCND) database, and the National Climatic Data Center (NCDC) TD3200 and TD3210 station data were used for the United States. Relative humidity and wind speed were generated using the Global Summary of the Day (GSOD) daily gridded data. The 10-km gridded data of solar radiation were produced using coarser resolution NCEP/NCAR reanalysis 1 data [47] and the NCEP/DOE AMIP 2 reanalysis data [48]. Hourly variations in climatic variables are simulated through the use of empirical formulations of temperature, specific humidity, precipitation, and radiation variability [29].

Land surface inputs at model initialization include soil textural class at each soil layer to a depth of 250 cm. The dominant soil texture for each soil layer in each 5-minute grid cell was derived from the USDA State Soil Geographic Database (STATSGO) 1 km resolution dataset [49]. The standard thicknesses of the 11 soil layers are 5 cm (layers 1 and 2), 10 cm (layers 3–5), 20 cm (layers 6–8), and 50 cm (layers 9–11), coinciding with the CONUS-Soil dataset. From the assignment of a textural category in each grid cell and each soil layer, the porosity, field capacity, wilting point, saturated air-entry potential and hydraulic conductivity, and moisture release curve “b” (Campbell) coefficient are obtained from a look-up table [29]. Soil moisture is used in combination with snow and vegetative properties to determine the land surface albedo in the absence of surface residues.

### Implementation

The geographic region delineated for this study was between −79W and −105W longitude, and 35N to 50N latitude, excluding portions of Canada. While we carry out simulations of miscanthus growing everywhere across this region, this is done purely as a scientific exercise and not as a specific recommendation. We performed multiple simulations to (1) validate simulations of snow depth and 10 cm soil temperature against historical observations, and (2) examine the effects of changing land cover and management on annual minimum wintertime soil temperatures deemed critical to miscanthus rhizome overwintering, and how those temperatures are impacted by differing soil surface residue thicknesses, laying prostrate and evenly distributed (Table 1). Simulations represented potential (natural) vegetation (POTVEG); maize managed with conventional tillage (MAIZE+TILL) leaving no surface residue or stubble post harvest; maize managed with no-tillage (MAIZE+NOTILL) that left a 5 cm thick surface residue layer post harvest, but no standing stubble; *M. × giganteus* with an annual fall harvest leaving varying thicknesses of surface residue (1.0, 2.5, and 5.0 cm), with the intent to have a thatch layer present during each subsequent winter and spring, (MISCAN+R; Table 1). The prescribed range of residue thicknesses for miscanthus used in regional simulations are consistent with observations [50].

**Table 1:** Description of Agro-IBIS model runs.

Model Simulation	Description	Residue Layer?
POTVEG	Potential vegetation representing natural vegetation types that could grow in each grid cell based on bioclimatic limits of each plant functional type; dynamic vegetation modeling	No
MAIZE+TILL	Continuous maize managed with conventional tillage; fall harvest removes all aboveground vegetation, leaving a bare soil surface	No
MAIZE+NOTILL	Continuous maize managed with no-tillage; fall harvest management leaves dry plant matter on field with an assumed thickness of 5 cm.	Yes
MISCAN+R_1 cm_	Miscanthus grown each year; fall harvest management leaves dry plant matter on field with an average thickness of 1 cm.	Yes
MISCAN+R_2.5 cm_	Miscanthus grown each year; fall harvest management leaves dry plant matter on field with an average thickness of 2.5 cm.	Yes
MISCAN+R_5 cm_	Miscanthus grown each year; fall harvest management leaves dry plant matter on field with an average thickness of 5 cm.	Yes

Given the focus of this study on regional soil temperatures and the important connection between snow depth and wintertime minimum soil temperatures [22], [51], we reassessed Agro-IBIS simulations of soil temperature and monthly and seasonal snow depth across the Midwest US. Numerous studies have discussed the insulating properties of snowpack, thereby decreasing the depth of frost penetration, as well as the difficulty in modeling the transition from snowpack to a existence of a bare soil surface during spring in temperate latitudes [52], [53]. We first used snow depth and soil temperature datasets previously constructed by Lenters et al. [30] and Hu and Feng [54], respectively. Iowa soil temperatures from the Hu and Feng [54] dataset were selected for model validation because of the high number of soil temperature observations available from 1982–2002. The majority of stations in the Hu and Feng [54] dataset had soil temperature readings made beneath soils void of vegetative or residue cover but could not always be confirmed.

We also used additional sources of soil temperature data at agricultural research sites to further validate Agro-IBIS. First, simulated 10 cm soil temperatures for the 2009–2011 period were compared to 10 cm soil temperature data (chromel-constantan thermocouples) collected in fields managed for conventionally tilled maize and switchgrass with a sparse thatch layer at the University of Wisconsin-Madison Arlington Agricultural Research Station, near Arlington, WI (43.31°N Lat, −89.38°W Lon). Next, we used observed 10 cm soil temperatures (Hydra Probe II, Stevens, Portland, OR USA) for the 2009–2011 period collected in five *M. × giganteus* plots at the University of Illinois Energy Farm (40.06°N Lat, −88.20°W Lon; for full site and plot description see Zeri et al. [55] and Smith et al. [56]). For this comparison, the model was driven with a climate data set developed specifically for the University of Illinois Energy Farm [24], [25]. Lastly, we compiled 10 cm soil temperature data from 125 observation sites in the Midwest US – across eleven states – that have collected continuous 10 cm daily soil temperature data during a portion of the years 1981 through 2011 (SI, Table S1). Given our inability to exactly replicate specific site management history, soil profiles, and hourly meteorological conditions at each of the 125 observation sites in this comparison, we used a more conservative 3-day running mean of 10 cm soil temperatures in Agro-IBIS to estimate the simulated extreme minimum soil temperatures, and to compare with the daily extreme minimums measured at each site. These station data were used to assess the ability of Agro-IBIS to simulate the average annual extreme minimum 10 cm soil temperatures across a large geographic extent, and the frequency that the 10 cm soil temperature falls below −3.5°C and −6°C, respectively. The majority of stations had soil temperature readings made beneath soils void of vegetative or residue cover with the exception of a subset of the 125 sites located in Illinois, which had grass cover (Carl Bernacchi, personal communication).

The POTVEG, MAIZE+TILL, and MAIZE+NOTILL model simulations were used to validate monthly and seasonal (Nov-Apr) averages of snow depth for the 1963–1995 time period [30] and monthly 10 cm soil temperatures from 1980–2002 across Iowa [54]. For validation of simulated monthly and seasonal snow depth, we used the same observational snow depth dataset from the National Weather Service Summary of the Day that was used in a previous validation of the IBIS-2 model by Lenters et al. [30]. Agro-IBIS spatial averages were formed for all grid cells in northern quadrants (grid cells within 43.5° to 47.5°N lat and −94.0 and −83.0°W lon) and southern quadrants (from 39.5° to 43.49°N lat and −94.0° and −83.0°W lon) to compare with averages for 34 station observations. The simulation of extreme minimum soil temperatures was validated using the daily average 10 cm soil temperature output from the MAIZE+TILL simulation was compared with minimum temperature data obtained from the 125 sites (SI, Table S1).

Regional simulations were conducted from 1940 to 2007. However, because of changes in climate across the region [57], [58], we limited our historical analyses of minimum 10 cm soil temperatures and frequency of occurrences to a shortened 30-year period from 1978–2007. The first eight years of the 1940–2007 simulations were discarded as spin-up, needed to bring the soil water balance into equilibrium. We drove those eight years with randomly selected years of climate data. Simulations assumed that nitrogen (N) was not a limiting factor to plant growth. For simulations of maize, yearly changes in optimal planting dates and cultivar selection (total growing degree days required to physiological maturity) were simulated. All simulations were performed with a static atmospheric CO_2_ concentration of 370 ppm.

The Midwest US has experienced significant warming temperatures during the past several decades [57], [58], particularly during winter and springtime. Given these changes, we also investigated whether soil temperatures have experienced similar warming, thereby lowering the risk of unsuccessful overwintering of miscanthus. We used Agro-IBIS and our daily climate dataset from 1948–2007 to analyze trends in annual average soil temperatures, as well as changes in the extreme minimum values at a 10 cm depth. We also analyzed trends in the annual average soil temperatures at three depths in the MAIZE+TILL simulation from 1967–2002 to coincide with the Hu and Feng [54] study for comparison. All statistics were performed off-line using a commercial software package [59]. To be considered statistically significant, trends had to differ from zero at P<0.05.

### Simulating the effects of residue in Agro-IBIS

For the MAIZE+NOTILL and the three MISCAN+R regional simulations, the top model soil layer (0–5 cm for MAIZE+NOTILL, and varying thicknesses for MISCAN+R; Table 1) was modified to represent an organic residue layer, lying prostrate, that persisted throughout the year. The variables modified to represent a thatch layer and the values for key maize and miscanthus residue properties are presented in Table 2. We also used several additional sensitivity analyses at eight geographic locations that experience a wide range in average wintertime soil temperatures and snowfall. These additional model runs were used to further investigate the impact of (1) a wider range of ten different miscanthus straw residue thicknesses (from 1 cm to 20 cm), (2) varying residue albedo (from 0.15 to 0.50), and (3) porosity (bulk density) of residue material (from 0.5 to 0.99) on annual average extreme minimum soil temperatures. The ten additional miscanthus residue thickness simulations were performed to develop more easily interpreted response curves (e.g., soil temperature warming response to residue thickness), as well as to investigate how changing residue thicknesses impacts interannual variability in minimum soil temperatures. These types of responses would be difficult to illustrate succinctly with a series of spatial maps. We note that crop residue thicknesses greater than about 10 cm should be considered extreme scenarios that were used solely for the purpose of building response curves, and are not an easily implemented or recommended residue management option. In the case of miscanthus residue albedo and porosity, there is currently very little published data concerning values for these variables. In order to understand whether our simulation results could be biased due to choosing a mean value for miscanthus residue albedo and bulk density, we further investigated whether large changes in these quantities can have a significant impact on soil temperature responses associated with residue management.

**Table 2:** Plant residue biophysical values used to modify Agro-IBIS to simulate the effects of crop residue on soil surface energy balance and heat transfer.

Quantity	Maize residue	Reference	Miscanthus straw	Reference
Residue layer thickness (m)	0.05	[18]	0.022–0.042	[50]
Roughness length (m)	0.012	[76]	0.0065	[77]
Bulk density (kg m^−3^)	36.4	[78]	22.0	[50]
Cellulose density (kg m^−3^)	1450	[18]	1350	[79]
Thermal conductivity (W m^−1^ K^−1^)	0.126	[80]	0.08	[81]
Specific heat (J kg^−1^ K^−1^)	1900	[82]	1335	[81]
Porosity	0.975	[78]	0.96	[83]
Albedo	0.25	[84]	0.32	[85]
Fractional cover	0.95	[18]	0.90	Kucharik (unpublished data)

## Results

### Validation of simulated snow depth

**Figure 1:**
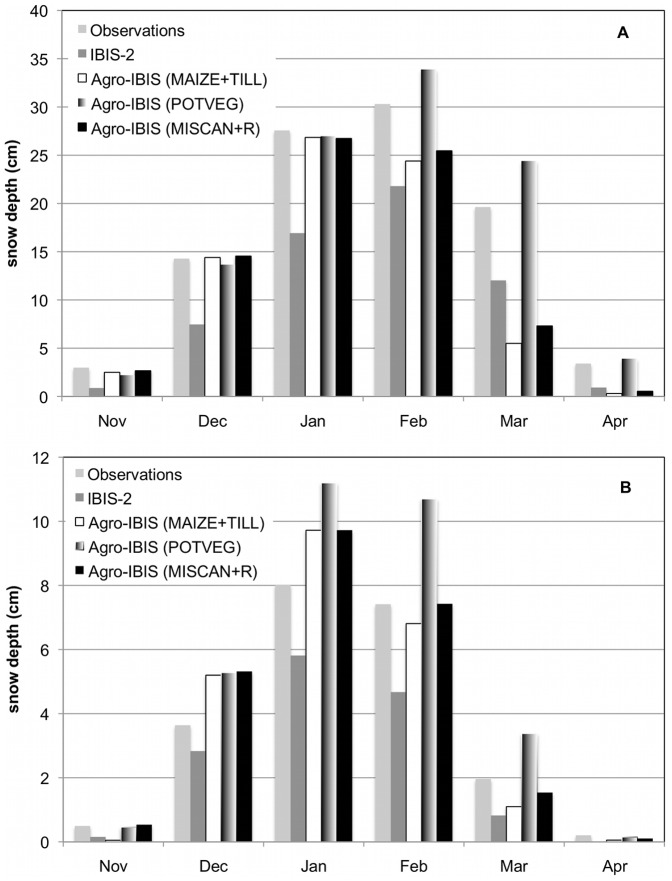
Comparison of observed and simulated monthly mean snow depths. Long-term monthly mean snow depths (1963–1995) for 34 Midwest station observations compared to previous IBIS and current Agro-IBIS simulations in (a) northern (43.5° to 47.5°N lat; −94.0 and −83.0°W lon) and (b) southern (39.5° to 43.49°N lat; −94.0 and −83.0°W lon) areas where observation stations were located. Observations and IBIS-2 results were obtained directly from previous statistical analyses for comparison here (Lenters et al., 2000).

Observed monthly mean snow depth in the northern region illustrated gradual increases from November through February, with an average maximum of approximately 30.3 cm occurring in February, declining to 19.6 cm in March and 3.4 cm in April (Fig. 1a). Compared with a previous version of IBIS that was executed over the Midwest with different climate and soils datasets at coarser spatial resolution [30], Agro-IBIS simulations exhibited increased snow depth in all months from December through March for the POTVEG scenario, which is also how land cover in Lenters et al. [30] was parameterized. In the POTVEG scenario, simulated snow depths were within ±5–10% of observations in all months from November through April, and the model captured the timing of the observed seasonal maximum snow depth in February. Model simulations for MAIZE+TILL scenario also showed higher simulated monthly mean snow depth from December through February compared to previous IBIS-2 simulations, but were approximately 15% and 75% lower than observed averages for February and March, respectively, and simulated maximum values occurred in January (Fig. 1a).

Observations of mean snow depth in the southern region illustrated gradual increases in monthly values from November through January, with an average maximum of approximately 8.0 cm occurring in January, declining to 7.4 cm in February and 2.0 cm in March (Fig. 1b). Compared to the previous IBIS-2 model results for a POTVEG scenario [30], simulations exhibited increased snow depth in all months for a similar vegetation parameterization and were 10–50% (i.e. 1–4 cm) higher than observed values in all months but matched the observed January maximum (Fig. 1b). Model simulations for MAIZE+TILL scenario also suggested improved simulated monthly mean snow depth from December through February compared to previous IBIS-2 simulations, were within 2 cm of observed values from December through March, and correctly simulated the timing of the observed snow depth maximum in January (Fig. 1b).

**Figure 2:**
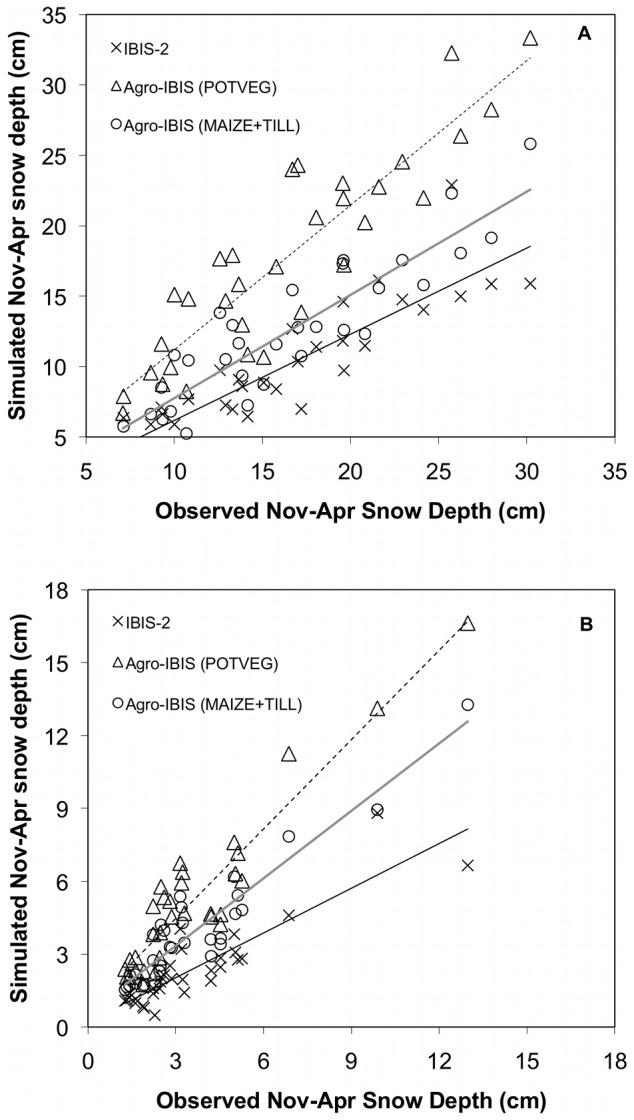
Observed versus simulated interannual variability of mean Nov-Apr snow depth. Comparison of observed versus Agro-IBIS model simulated mean Nov-Apr snow depth for each year from 1963–1995 in (a) northern (43.5° to 47.5°N lat; −94.0 and −83.0°W lon) and (b) southern (39.5° to 43.49°N lat; −94.0 and −83.0°W lon) areas where observation stations were located. Previous IBIS-2 model results (Lenters et al., 2000) are plotted for comparison. Linear regression fits between observations and model results are denoted by the following lines: thin black (IBIS-2); gray solid (MAIZE+TILL); black dashed (POTVEG).

In the current study, all three scenarios showed significant improvement over the previous IBIS-2 model validation (slope = 0.61; *r^2^* = 0.78) across the northern region (Fig. 2a). Both cropping system scenarios resulted in a negative bias and underestimated mean annual snow depth (slope  = 0.73; *r^2^* = 0.81 for MAIZE+TILL), while the POTVEG scenario closely captured the observed mean annual snow depth for each year (slope = 1.02, *r^2^* = 0.82). In the southern region (Fig. 2b), all three scenarios plotted showed improvement over the previous IBIS-2 model validation (slope = 0.61; *r^2^* = 0.79). Both cropping system scenarios resulted in close approximations to mean annual snow depth and simulated interannual variability well (slope  = 0.93; *r^2^* = 0.88 for MAIZE+TILL and slope  = 0.99), while the POTVEG scenario generally overestimated the observed mean annual snow depth (slope  = 1.23), but captured interannual variability in snow depth as well as the cropping system simulations (*r^2^* = 0.88).

### Validation of simulated monthly soil temperatures

**Figure 3:**
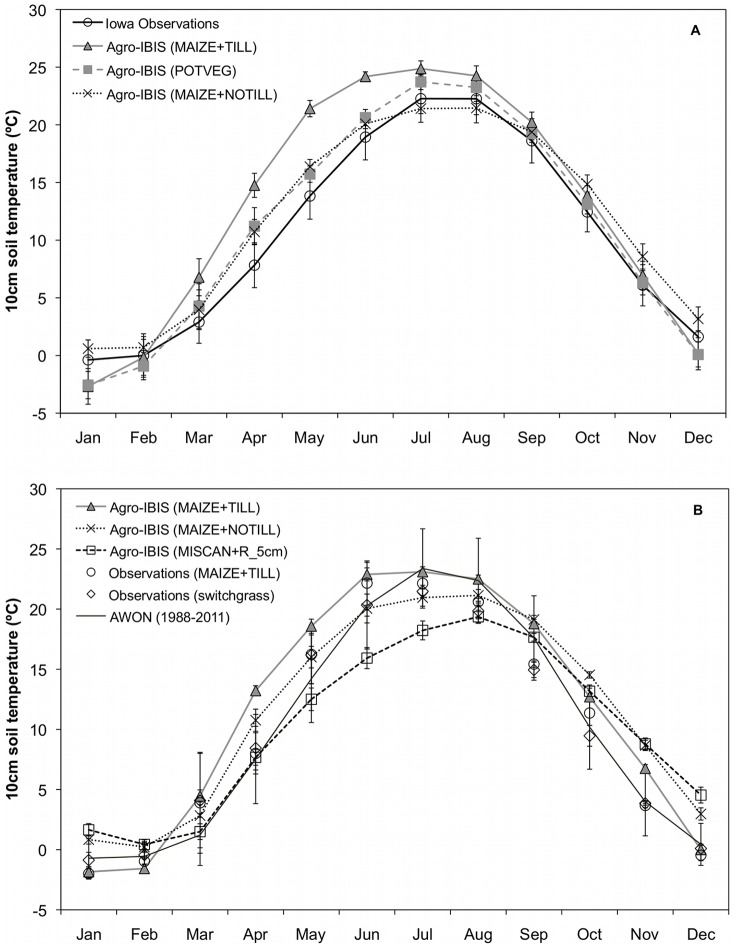
Comparison of observed and simulated soil temperatures in Iowa and Wisconsin. (a) Comparison of monthly average 10 cm soil temperatures for three Agro-IBIS model simulations with observational station data from Hu and Feng (2003) averaged over the state of Iowa for 1982–2002. Error bars are ±1 S.E. for both simulated and observed values. (b) Comparison of monthly average 10 cm soil temperatures for three Agro-IBIS models simulations compared with observational data at the University of Wisconsin-Madison Arlington Agricultural Research Station in tilled maize and switchgrass study plots for July 2009- June 2012. Observed data (maize and switchgrass) represent the monthly mean among 3 replicate plots (n = 3). Long-term averages (1988–2011) of monthly mean 10 cm soil temperature collected at the UW-Madison Automated Weather Observing Network (AWON) site at Arlington are plotted for comparison. Error bars are ±1 S.E. for all simulated and observed values.

The MAIZE+TILL simulations had a warm bias compared to the Iowa observations of about 3 to 8°C from March to June (Fig. 3a), which corresponds with the bias of an early spring snowmelt across the northern regions of the study area (Figs. 1, 2). The MAIZE+NOTILL and POTVEG simulations were in much better agreement with observed values across Iowa, although the MAIZE+NOTILL model runs showed a warm bias from September to January, and January is the month when the coldest monthly average temperatures occur (Fig. 3a). The MAIZE+TILL simulation exhibited a small cold bias in the December-February time period of about 0.25 to 3°C.

Simulated soil temperatures at the University of Wisconsin-Madison site showed a similar pattern to the findings in the state of Iowa for the two maize simulations (Fig. 3b). There was a general model warm bias in early spring to early summer of 2–7°C for the MAIZE+TILL simulation (bare soil), very similar to observations from June through December, and a slight cold bias of about 1°C in January. However, the presence of the 5 cm thick residue layer in both the MAIZE+NOTILL and MISCAN+R_5 cm_ simulations caused soil temperatures to have a cool bias of about 1–6°C during the peak of the summer and then remain warmer than typical observed values from October through February (Fig. 3b).

**Figure 4:**
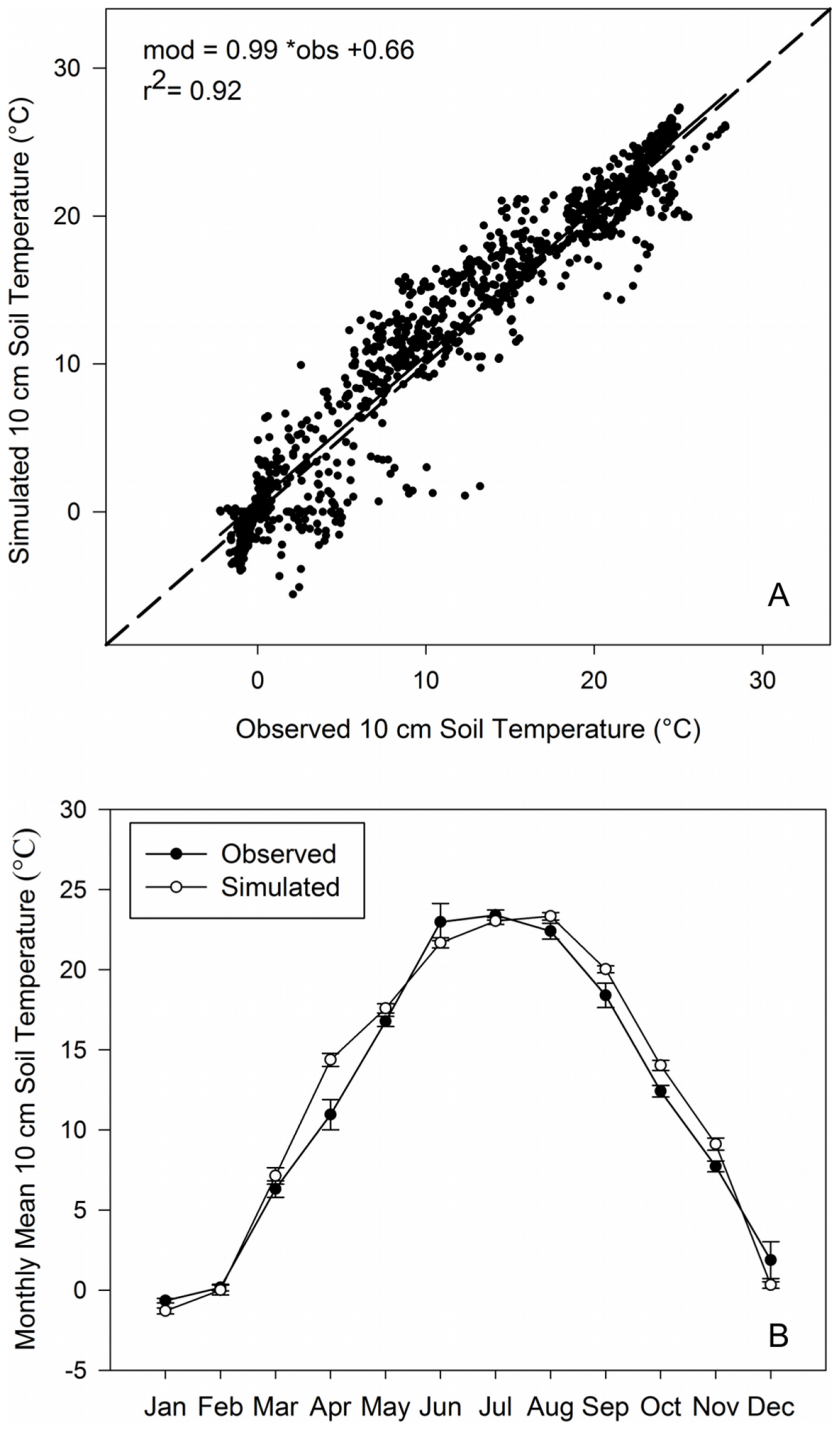
Comparison of observed and simulated soil temperatures in Illinois. (a) Comparison of 3-day running mean 10 cm soil temperatures for Agro-IBIS model simulations of miscanthus with no residue layer with observed values at the University of Illinois Energy Farm from 2009 to 2011. Observed values are the daily mean (n = 1 to 5) of observation for the miscanthus plots, n was less than five for periods when sensors were damaged, with n at least 3 for 80% of the days. (b) Comparison of monthly mean 10 cm soil temperatures for the same site and simulation. Data points are the mean (n = 3) of the 2009–20011 monthly values, error bars are ±1 S.E. for both simulated and observed values.

Simulated 3-day running mean 10 cm soil temperatures at the University of Illinois Energy Farm site for miscanthus without a residue layer present compare well with observations (Fig. 4a; *r^2^* = 0.92, P<0.0001) with a slope of 0.99 (S.E. 0.008) and intercept of 0.66 (S.E. 0.126). There were relatively large model errors at the freezing point, however, simulated values are typically within 2.5°C when observed temperatures were less than −1°C. At the monthly time scale, simulated values were ±1.7°C of the observed values for all months except April, and within 1.6°C during the winter months (December, January, February; Fig. 4b). Overall simulated values showed no consistent bias relative to observations, however, there was a slight underestimate (<0.8°C) of monthly mean 10 cm soil temperature during winter months.

### Validation of simulated annual extreme soil temperatures

**Figure 5:**
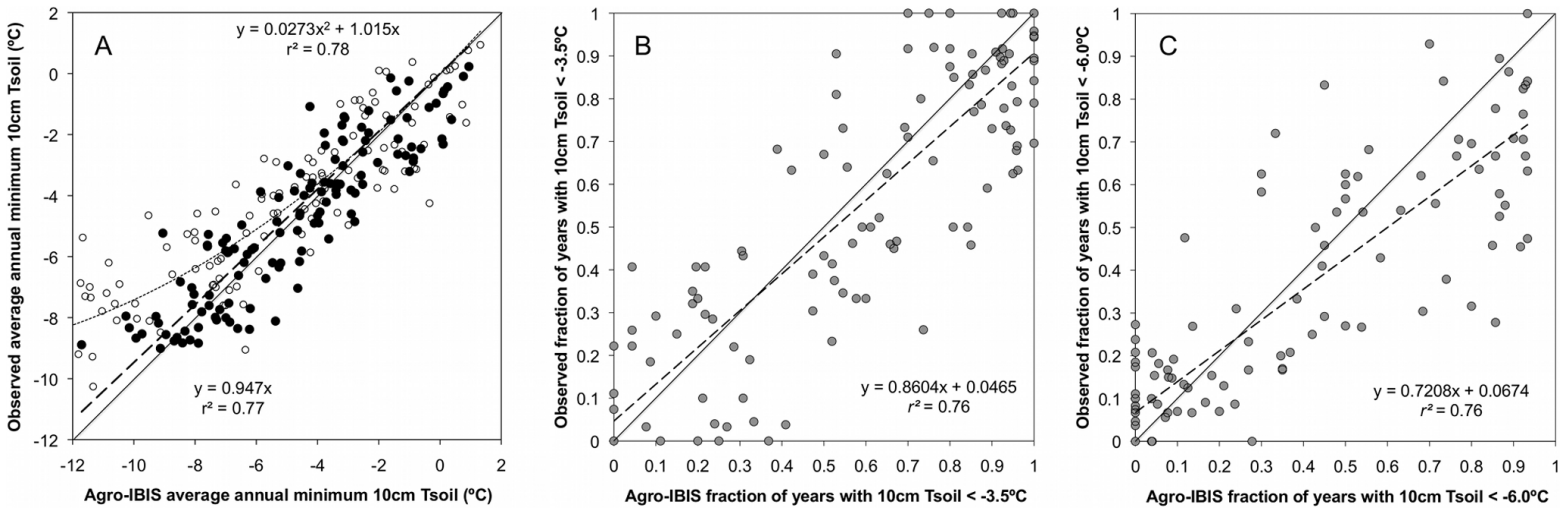
Comparison of observed and simulated annual extreme minimum 10 cm soil temperatures. (a) Agro-IBIS average annual extreme minimum 10 cm soil temperature (based on 3-day running mean temperatures) compared with observations from 125 observation sites (SI, Table S1) from the Midwest USA for original model values (open circles and dotted regression line), and statistically adjusted model values (filled circles and dashed regression line); (b) Agro-IBIS simulated frequency of occurrence of 10 cm soil temperatures (3-day running mean) reaching −3.5°C and (c) −6.0°C, respectively, compared with results from 125 observation sites in the Midwest USA. In these comparisons, Agro-IBIS was only simulated for a period from the beginning year that data was available for each observation station through 2007, which denotes the last year that gridded daily climate data was available.

Overall, Agro-IBIS performed exceedingly well for extreme minimum temperatures greater than approximately −6°C, but there was an increasing cold bias in model simulations as average annual observed extreme minimum temperatures decreased from about −6°C to −12°C (Fig. 5a; *r^2^* = 0.78, P<0.0001). For example, the simulated cold bias was about −0.5°C at −6°C (observed temperature), and −2°C at −8°C. The validation exercise suggested that a 2^nd^ degree polynomial model fit best approximated the relationship between simulated and observed average annual extreme minimum 10 cm temperatures (Fig. 5a).

Before comparing simulations to the observed fraction of years below the −3.5°C and −6°C thresholds, simulated annual extreme minimum soil temperatures were adjusted based on the regression analysis from the validation exercise (Fig. 5a). The simulated annual average minimum 10 cm soil temperatures had the mean bias removed by adjusting simulated values using the mean response relationship (2^nd^ degree polynomial) between observed and simulated quantities and its numerical deviation from a linear relationship with a slope  = 1.0. The model corrected data, also shown in Fig. 5a, exhibited greatly improved agreement, particularly at the coldest soil temperatures (*r^2^* = 0.77, P<0.0001, slope  = 0.947). This statistical adjustment process did not significantly affect our ability to capture the soil temperature variability among observational sites, denoted by the similar *r^2^* values (0.78 vs. 0.77) for the original and corrected model fits (Fig. 5a). The linear model fit through the observed data and model simulated output for frequency of occurrence of −3.5°C temperatures (Fig. 5b) resulted in an *r^2^* = 0.76 (P<0.0001), with a slope of 0.86 (S.E. 0.043) and intercept of 0.046 (S.E. 0.029). For the frequency of occurrence of −6.0°C temperatures across the region (Fig. 5c), the linear model fit through the observed data and model simulated output resulted in an *r^2^* = 0.76 (P<0.0001), with a slope of 0.72 (S.E. 0.036) and intercept of 0.067 (S.E. 0.018).

### Average annual extreme minimum 10 cm soil temperatures: 1978–2007

**Figure 6:**
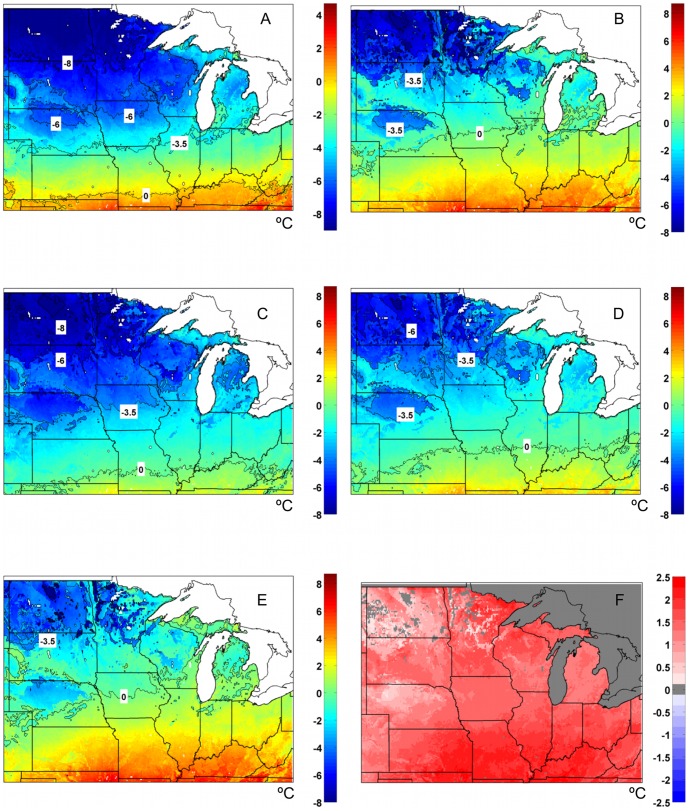
Impacts of soil surface residue management on annual average extreme minimum 10 cm soil temperatures. Average annual (1978–2007) extreme minimum 10 cm soil temperatures (based on 3-day running mean) for (a) MAIZE+TILL (bare soil post harvest), (b) MAIZE+NOTILL, (c) MISCAN+R_1 cm_, (d) MISCAN+R_2.5 cm_, and (e) MISCAN+R_5 cm_ simulations. The position of the 0.0°C, −3.5°C, −6.0°C, and −8.0°C 10 cm soil temperature isopleths are highlighted by labels on solid black lines; (f) differences in average annual extreme minimum 10 cm soil temperatures for MISCAN+R_5 cm_ – MAIZE+NOTILL (Fig. 6e–Fig. 6b results).

Analysis of the average annual extreme minimum soil temperatures at a 10 cm depth for the control bare soil case (MAIZE+TILL) model simulation suggest that an absence of any residue layer after fall crop harvest would result in the majority of the Midwest region commonly experiencing temperatures below 0.0°C each year (Fig. 6a). The −3.5°C and −6°C thresholds are generally reached over smaller regions of the Upper Midwest, but are still present in core areas of the Corn Belt. The coldest average annual extreme soil temperatures at 10 cm are in the −8°C to −11°C range confined to large portions of North Dakota and Minnesota (Fig. 6a). However, the MAIZE+NOTILL simulations (Fig. 6b) suggest that a 5 cm thick, continuous cover of maize residue helps to provide a widespread insulating effect on minimum soil temperatures. Most of the region experienced a warming of the extreme minimum temperatures from 2.5°C to 6°C for MAIZE+NOTILL compared to the bare soil (MAIZE+TILL) simulation. However, this analysis suggests regions that typically experience greater snowfall in the far northern portions (e.g., North Dakota and Minnesota), the upper peninsula of Michigan, and on the eastern side of lake Michigan, would not warm as much during the winter with a persistent 5 cm thick residue layer. The areas that saw the greatest warming impact of residue were located in the central portions of the Midwest, across northern Iowa, eastern South Dakota, southern and central Minnesota, and much of Wisconsin.

**Figure 7:**
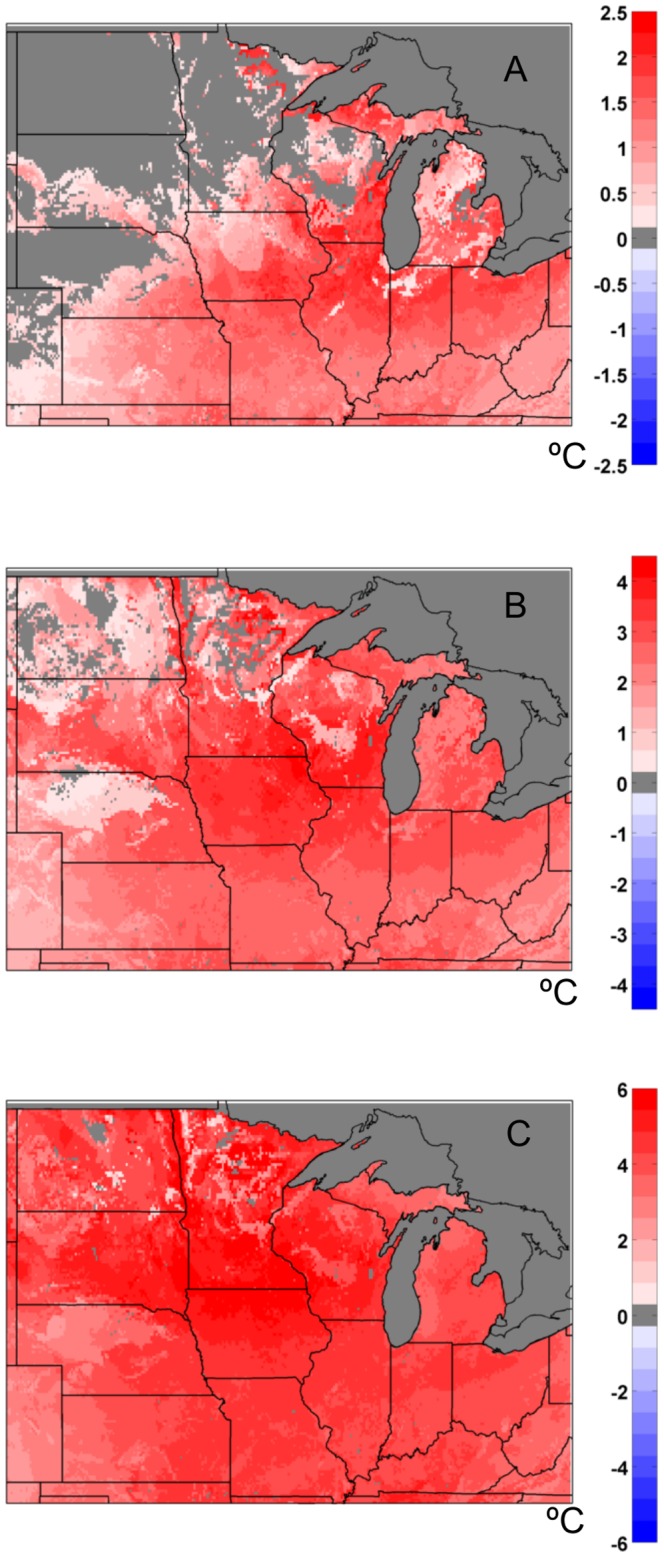
Soil temperature differences between different residue layer thicknesses. Average annual (1978–2007) extreme minimum 10 cm soil temperature differences, based on a 3-day running mean, for the following paired simulations: (a) MISCAN+R_1 cm_ minus MAIZE+TILL (bare soil post harvest), (b) MISCAN+R_2.5 cm_ minus MAIZE+TILL, (c) MISCAN+R_5 cm_ minus MAIZE+TILL.

Analysis of the average annual extreme minimum soil temperatures at a 10 cm depth for the series of miscanthus simulations with varied residue thicknesses (1, 2.5, and 5 cm) suggest that as residue thicknesses increase, the magnitude of the insulating effect on annual minimum soil temperatures also increases (Figs. 6c–6e). The residue simulations for the 5 cm thick residue layer for miscanthus (MISCAN+R_5 cm_; Fig. 6e) suggest that miscanthus straw has a slightly increased insulating effect than maize stover, given different biophysical properties (Table 2). Regionally, the largest difference between miscanthus straw and maize stover was in southern portions, with a ∼2°C increase in the extreme minimum temperatures while differences in the far northwest portion were typically less than 0.5°C (Fig. 6f). The impacts of a 1 cm thick miscanthus straw layer, compared to the bare soil scenario, were minimal over northwest portions of the region, where no warming of minimum soil temperatures occurred (Fig. 7a). More widespread warming of minimum soil temperatures of 0.5°C to as much as 6°C occurred with miscanthus residue thicknesses of 2.5 cm (Fig. 7b) and 5 cm (Fig. 7c), respectively, compared to the bare soil case. However, even in the 2.5 cm scenario, the magnitude of warming was minimal across far northwestern portions of the region (0°C to only 0.5°C), as well as areas in central Wisconsin and northcentral Nebraska that had soils with higher sand content (Fig. 7b). Thus, many areas still had annual average minimum 10 cm soil temperatures that were below −3.5°C and −6.0°C when miscanthus residue was less than or equal 2.5 cm thick (Figs. 6c,d). The largest magnitude of warming associated with both 1 cm and 2.5 cm miscanthus residue thicknesses was focused in a corridor from eastern Nebraska, through Iowa, southern Minnesota, northern Illinois and southern Wisconsin.

### Expanded investigation of varied miscanthus residue depth on minimum soil temperatures

**Figure 8:**
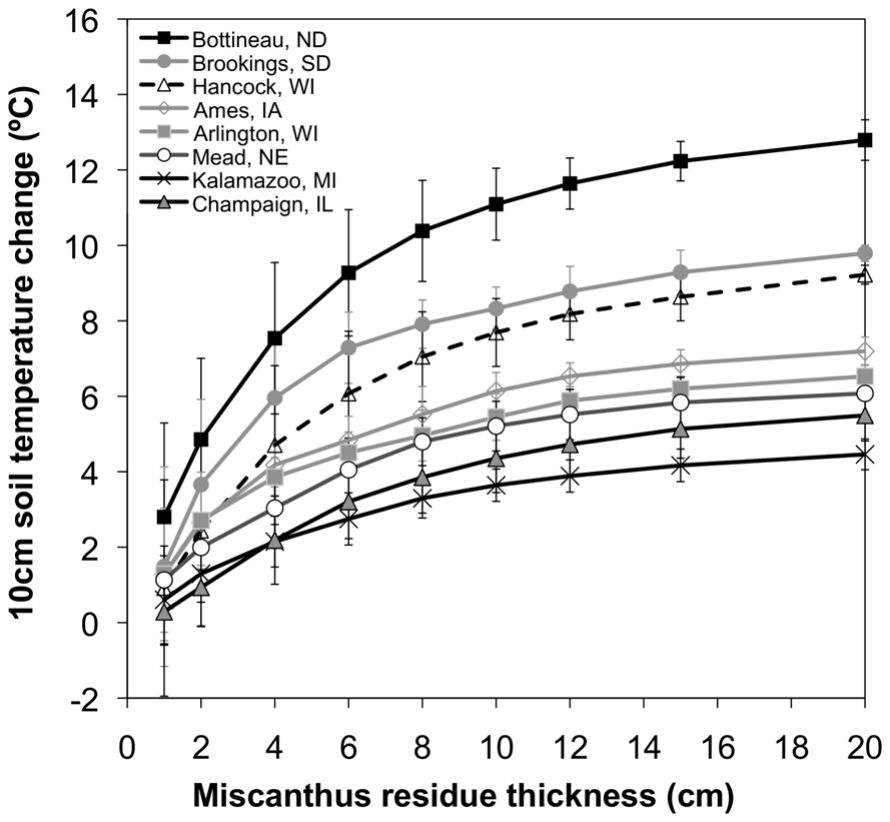
Impacts of miscanthus residue thickness on annual average extreme 10 cm soil temperatures. Average annual (1978–2007) extreme minimum 10 cm soil temperature changes at eight locations in the Midwest, based on a 3-day running mean, for MISCAN+R simulations with varied residue thicknesses relative to the MAIZE+TILL (bare soil) simulation.

The warming of annual average minimum temperatures with 20 cm of miscanthus straw residue could be as little as approximately 4°C, to as great as 12°C depending on geographic location (Fig. 8). Although the soil warming effects were maximized for a residue thickness of around 20 cm, the shape of the response curves suggests that even greater warming could occur with greater thicknesses regardless of location throughout the Midwest US (Fig. 8). However, we reiterate that residue thicknesses greater than approximately 10 cm are extreme scenarios and not realistic management options in the field. The 5 cm and 10 cm thick residue layers for miscanthus produced about 60% and 84%, respectively, of the warming benefit associated with the thickest residue cover simulated. Additionally, the interannual variability of the coldest 10 cm soil temperatures was reduced as residue thickness increased (Fig. 8).

### Likelihood of reaching critical minimum soil temperatures: 1978–2007

**Figure 9:**
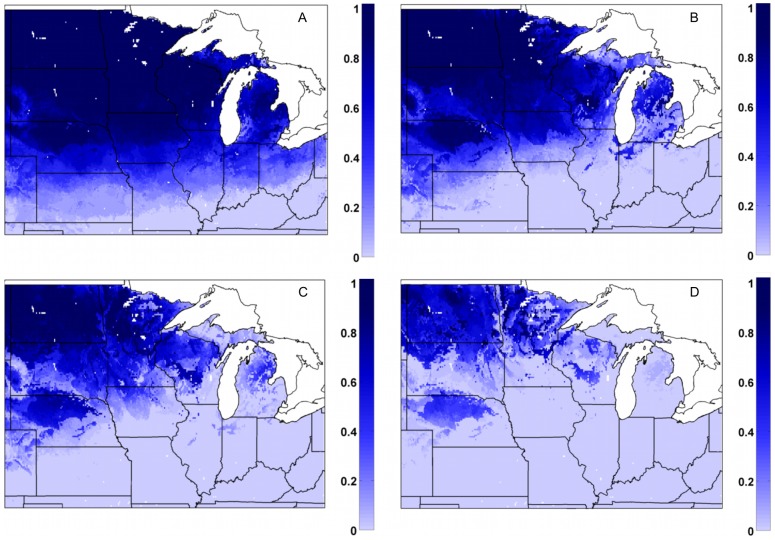
Frequency of 10 cm soil temperatures reaching −3.5°C or colder for varied miscanthus residue thicknesses. Fraction of total years during the 1978–2007 time period that simulated annual 10 cm soil temperatures were at or below a −3.5°C threshold (based on a 3-day running mean) for (a) MAIZE+TILL (bare soil), (b) MISCAN+R_1 cm_, (c) MISCAN+R_2.5 cm_, and (d) MISCAN+R_5 cm_ simulations.

In the bare soil control simulation (MAIZE+TILL), large portions of the upper Midwest would have experienced 10 cm soil temperatures below −3.5°C in 75–95% of the years, and the risk of those temperatures being reached is not completely eliminated unless fields are located in far southern regions (Fig. 9a). The risk for these cold temperatures is reduced for the three scenarios of varied miscanthus straw thicknesses, with a corresponding relationship between the magnitude of reduced probabilities and residue thickness (Figs. 9b–d). In these simulations, the likelihood of reaching −3.5°C was considerably lower over southern and eastern portions of the Midwest US, but across the Dakotas as well as portions of Nebraska, Minnesota, and Wisconsin, about 40–80% of years had 10 cm soil temperatures reaching −3.5°C even with a 5 cm residue layer (Fig. 8d). While the effectiveness of a 1 cm thick miscanthus residue layer was much lower, even this small amount greatly reduced the probability of reaching −3.5°C across large portions of Kansas, Missouri, Illinois, Indiana, and Ohio (Fig. 9b).

**Figure 10:**
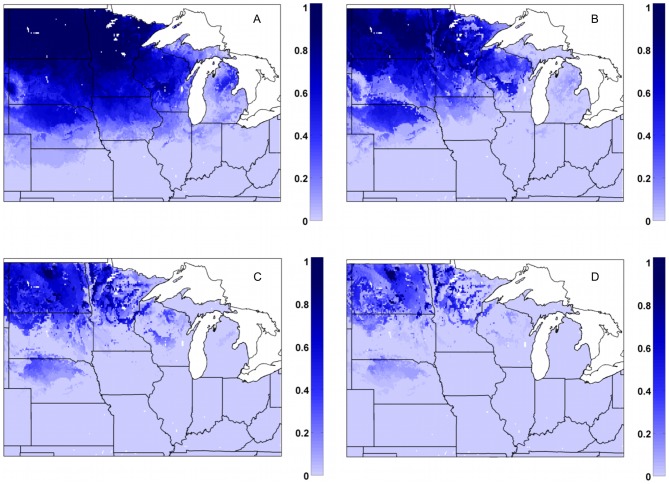
Frequency of 10 cm soil temperatures reaching −6.0°C or colder for varied miscanthus residue thicknesses. Fraction of total years during the 1978–2007 time period that simulated annual 10 cm soil temperatures were at or below a −6.0°C threshold (based on a 3-day running mean) for (a) MAIZE+TILL (bare soil), (b) MISCAN+R_1 cm_, (c) MISCAN+R_2.5 cm_, and (d) MISCAN+R_5 cm_ simulations.

In the bare soil control simulation (MAIZE+TILL), smaller portions of the upper Midwest would have experienced 10 cm soil temperatures below −6.0°C in about 75% of the years and large sections of the Dakotas, Minnesota, and Wisconsin, reached this threshold in 50 to 60% of the years (Fig. 10a). The risk for these very cold temperatures was greatly reduced, but not completely eliminated, in the three miscanthus simulations (Figs. 10b–d). Similar to the results presented in Figure 9, the likelihood of reaching the −6.0°C threshold is reduced the most with a 5 cm residue layer (Fig. 10c), and the least in the 1 cm thickness scenario (Fig. 10b). However, even in the 5 cm simulation, the −6.0°C threshold was reached in 60–90% of all years in far northern regions of North Dakota and Minnesota (Fig. 10d).

### Impact of varied miscanthus residue albedo and bulk density on minimum soil temperatures

In general, variations in residue albedo or bulk density had small impacts on the simulated annual minimum soil temperatures. For residue thicknesses of 1 cm and 5 cm, a change in residue albedo from 0.15 to 0.5 contributed to minimum soil temperatures at 10 cm that were 0.3°C to 0.6°C colder. Over a more realistic range of likely albedo values (0.2 to 0.4), the contribution was only 0.1 to 0.2°C, or about 5–10% of the mean annual minimum 10 cm soil temperatures for the two residue thicknesses simulated in this sensitivity study. Bulk density values were varied, with particle (cellulose) density held fixed, to generate a range in porosity of the 1 cm and 5 cm layers from 0.5 to 0.99. Here, the net effect on minimum soil temperatures was even less than for albedo, contributing to a net change of 0.01–0.05°C.

### Soil temperature trends

Our regionally averaged results for soil temperature trends (e.g., a spatial average for the entire study region) produced values equal to 0.250°C (10 yr)^−1^ at 10 cm, 0.252°C (10 yr)^−1^ at 60 cm, and 0.253°C (10 yr)^−1^ at 100 cm during 1967–2002 for the MAIZE+TILL simulations (data not shown). We also compared observed trends in the annual extreme minimum 10 cm soil temperatures for station observations in our study (SI, Table S1) with the average simulated response at a subset of those sites that had continuous records of at least 27 years. The observed trend in annual extreme minimum 10 cm soil temperature, averaged across all 36 stations, was 0.88°C (10 yr)^−1^ (S.D. 0.097) compared to 0.78°C (10 yr)^−1^ (S.D. 0.030) for Agro-IBIS simulations.

**Figure 11:**
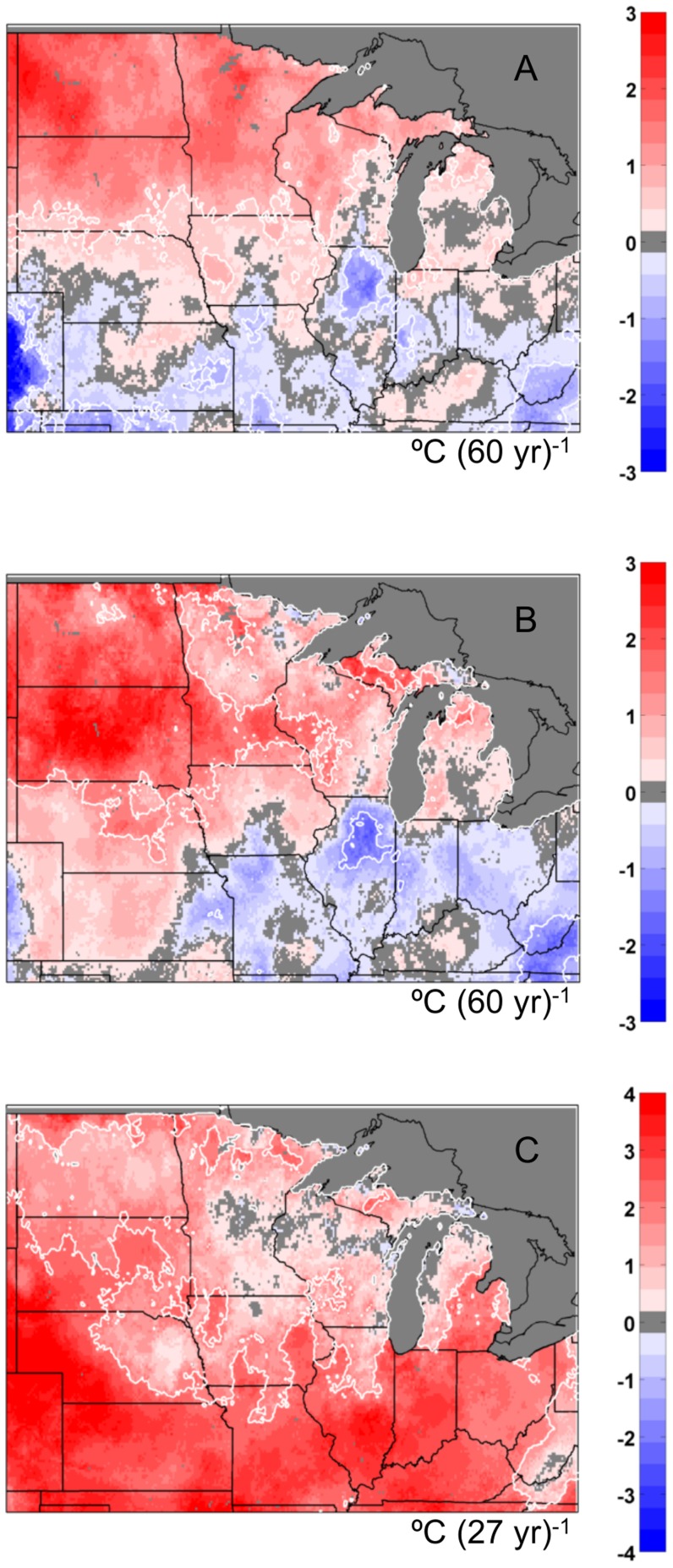
Simulated soil temperature trends across the Midwest US. (a) Total change (from linear regression) in annual average 10 cm soil temperatures for 1948–2007 for the MAIZE+TILL (bare soil) simulation; (b) total change (from linear regression) in annual extreme minimum 10 cm soil temperature for the MAIZE+TILL (bare soil) simulation from 1948–2007; (c) total change (from linear regression) in annual extreme minimum 10 cm soil temperature for the MAIZE+TILL (bare soil) simulation from 1981–2007. Regions bounded by solid white lines indicate trends with P<0.05.

The overall trend (°C per 60 years) in 10 cm annual average soil temperature varied widely from 1948–2007 (Fig. 11a). Some regions of the Midwest experienced significant cooling in contrast to warming of about 1–3°C. For the entire 1948–2007 period our analysis produced regionally averaged trends that were much lower than those for the 1967–2002 period; 0.059°C (10 yr)^−1^ at 10 cm; 0.052°C (10 yr)^−1^ at 60 cm, and 0.049°C (10 yr)^−1^ at 100 cm. There are similar spatial patterns in the trends in the annual extreme minimum 10 cm soil temperatures from 1948–2007, with the most significant warming of about 2–3°C having taken place in the northern Plains (Fig 11b). However, a dramatically different spatial pattern emerges when the analysis of trends is limited to the 1981–2007 time period (Fig 11c). Here, southern regions appear to have experienced the most significant warming up to 3–4°C of change in the annual extreme minimum temperatures, with a smaller magnitude of change across more northern states. The regionally averaged annual average 10 cm soil temperature trend was 0.33°C (10 yr)^−1^ for 1981–2007, and the spatial patterns of change across the Midwest were similar to trends in extreme minimum temperatures.

## Discussion

This study has described a dataset for annual extreme minimum temperatures across the Midwest US for both soils that are managed without leaving residue on the soil surface after harvest of crops as well as for soils that have a full cover, 5 cm thick prostrate residue layer for maize in place, and for varying thicknesses of miscanthus straw. Our results indicated that strategic residue management in the region has potential to help increase extreme minimum soil temperatures that can threaten overwintering of miscanthus rhizomes in the first year of establishment and potentially in later years. Based on previous research that has investigated the ability of winter wheat to survive extremely cold winters in the central US [60], it appears that a combination of leaving behind standing stubble after harvest to preferentially trap snow, coupled with a prostate residue layer, offers the highest likelihood of insulating soils and to increase the odds that miscanthus rhizomes can survive the first winter after establishment. However, as our results have shown, the thickness of that residue layer has significant bearing on the insulating effect.

### Influence of residue, soils, snowpack, and management on minimum extreme soil temperatures

The Midwest US is subjected each year to rapid and extreme temperature changes. However, due to the large presence of human management of agricultural lands, as well as differences in the timing of snowfall and the buildup of a consistent snow cover each year, air temperatures should not be perceived as a guide for estimating extreme minimum soil temperatures near the surface. For example, while air temperatures across regions of the Midwest can drop to −25°C to −40°C during the winter for extended periods of time, the absolute coldest soil temperatures at 10 cm were about −12° to −16°C, based on both observed data and simulated results for individual years in our study. According to observations in the region over the past 30 years, the average 10 cm minimum soil temperatures are in the range of −8°C to −10°C (Fig. 5a), which is much warmer than the typical average low air temperatures. This is attributed to the insulating effect of snow cover. The timing and duration of a consistent snow pack is highly influential in determining minimum soil temperatures in mid-January to early March, which is historically when soils reach their lowest temperatures [22], [51], [61].

Agro-IBIS simulations suggested that miscanthus straw could function better as an insulator than maize residue for a comparable 5 cm thick layer on the soil surface, which is largely attributed to differences in biophysical properties (Table 2). However, both plant residue types keep soils warmer during winter, thereby decreasing the risk of 10 cm soil temperatures going below −3.5°C over a large part of the Midwest. However, for decreased thicknesses of miscanthus residue (1 and 2.5 cm), the warming effect is considerably less, and in some locations across the Midwest, negligible. Our expanded sensitivity tests at eight locations concerning the impacts of a wide range of residue thickness on the magnitude of warming of the minimum soil temperatures suggested that 60% of the maximum warming benefit occurs with a 5 cm thick layer, and 84% with a 10 cm thick layer (Fig. 8). For 1 cm and 2.5 cm thick layers, only 15% and 40% of the maximum warming benefit occurs. While thicker residue layers clearly increase the warming benefit, data from field experiments (Table 2) suggest that a nominal thickness of only a few centimeters is likely easier to sustain across large fields given issues related to wind blowing loose residue around. We emphasize that the 20 cm residue thickness is an extreme scenario that was created to understand system behavior, and is not suggested as a recommended management practice.

The simulated soil warming attributed to residue in this study is in agreement with previous studies for an experiment in Minnesota that examined the effect of different maize residue management options on soil temperatures in the 0.05 to 0.3 m soil layer. Sharratt et al. [22] reported that wintertime minimum soil temperatures at a 1 cm depth were 5 to 8°C warmer over a three year period for a management scenario that left 60 cm of stubble standing in combination with residue laying prostrate on the soil surface compared to a treatment that had 0 cm stubble with all residue removed from the soil surface. While the magnitude of the warming effect associated with residue was greater in Sharratt et al. [22] then in our study, their temperatures were reported for a 1 cm depth. They also found that snow depth was influenced by the residue treatments, whereby fields that had stalks cut closer to the ground (e.g., 30 cm compared to 60 cm) had a lower average snow depth during the winter.

However, not all Midwest regions may see a significant soil warming from straw or maize residue due to the confounding influence of snow cover on winter soil temperatures. Model results illustrated that even with the addition of a 5 cm thick residue layer of either miscanthus straw or maize residue, this had minimal effects on the extreme minimum temperatures in small portions of North Dakota and Minnesota, and to a lesser extent the sandier soils of north and central Nebraska and the central sands region of Wisconsin (Figs. 6–7). This may be attributed to two reasons: first, across the most northern regions of the Midwest, snowfall comes earlier, and due to colder temperatures, snowpack depth and duration is generally greater and longer, respectively, than in more southern locations [62]. Sharratt et al. [63] suggested that approximately 15–42 cm of snow cover is needed to insulate the top portion of the soil profile from cold wintertime temperatures, resulting in near steady-state soil temperatures. Thus, the potential impact of a residue layer may be minimized in regions that historically have deeper and more consistent snow cover (e.g., North Dakota and northern Minnesota, as well as lake effect snow regions in Michigan), which might explain the simulated spatial patterns and results in our study. However, we note that in northern locations that typically have a significant, consistent snowpack during winter (e.g., >15 cm), observed and simulated 10 cm soil temperatures still reached well below 0.0°C; thus the timing of a building and retreating snowpack is also crucial, besides the thickness, in determining extreme minimum soil temperatures. Second, some of the spatial patterns of soil temperature change attributed to residue management suggest that soil texture plays an important role also in determining the magnitude of extreme minimum soil temperatures. In sandy soil regions, we hypothesize that a thicker residue layer may add a more prominent insulating effect on these soils that lose heat more rapidly due to their lower average volumetric water content in fall and inherent mineral properties [29].

These results suggest that in the first years of establishment of miscanthus, a soil surface residue layer could increase the probability of successful overwintering of the plant rhizomes, but the thickness of that layer is highly deterministic to the overall soil warming. This management option, coupled with leaving standing stubble that could preferentially trap snow, would likely provide the greatest likelihood of maximizing soil warming [22], [51]. However, during the first year or two of establishment when *M. × giganteus* might be the most susceptible to winterkill or damage to rhizomes, the amount of biomass produced may not be sufficient to support a residue layer thickness that significantly reduces the risk of lethal soil temperatures. Several studies from the literature suggest that *M. × giganteus* will take at least three years to reach the expected yield ceilings, and during the first year, annual productivity can typically be in the 1–4 Mg ha^−1^ range [4], [15], [64]–[66]. With a typical residue bulk density of 22 kg m^−3^ (Table 2), 2.2 Mg ha^−1^ of aboveground biomass is needed for each 1 cm of thatch depth. Thus, to achieve a 5 cm thatch thickness, approximately 11 Mg ha^−1^ of aboveground biomass would be required; these values may not be observed until year three and beyond [50], [55], [67]. Given these results, a producer might be faced with a new dilemma in the context of residue management. For example, a farmer may not have enough miscanthus biomass to sell to make a profit after the first year, so they would probably mow the crop. In the next two years, they will have to hedge the risk of maximizing profits vs. minimizing the cold temperature threat. Furthermore, they may face a more complicated decision on what to do with available residue from nearby corn fields; should it be used to build a solid residue layer to protect rhizomes?

Additional studies that quantify the relationship between miscanthus productivity, residue bulk density, and thatch thickness during early establishment years will help determine whether additional sources of crop residue from nearby fields (e.g., maize) are needed to build a more substantial protective layer to reduce the odds of winterkill. The research presented here showed that very small differences in thatch thickness lead to significant differences in minimum winter soil temperatures. Therefore, field trials should continue to be established that strategically manage residue in varied amounts on fields as well as investigate how stubble height and snow depth variations impact soil temperatures over several years to account for interannual variability. These data would also prove useful to help identify an optimal growing region in the US and Canada for miscanthus. Currently, there is also a lack of biophysical information on the properties of miscanthus straw (e.g., bulk density, albedo, heat capacity), which is needed to better constrain the parameterization of agroecosystem models.

### Soil temperature trends: are soils warming or cooling?

This study, as well as three other studies, have documented long-term changes in soil temperatures but arrived at different conclusions [54], [68], [69]. Using a limited number (38) of observation stations in the US that had a period of record from 1967–2002, Hu and Feng [54] reported that annual average 10 cm and 100 cm soil temperatures across these sites were increasing at a rate of 0.3°C (10 yr)^−1^, and the sites that had the greatest rate of warming were across northern regions. Sinha et al. [69] also reported warming soil temperatures at 10 cm during 1967–2006 in regions of Minnesota, Illinois, and Indiana, as well as a reduction in the number of days with soil frost in the Midwest. In contrast, Isard et al. [68] used a biophysical modeling approach and reported that even though wintertime air temperatures from 1951–2000 were increasing, wintertime soil temperatures at 50 cm depth were decreasing across the Great Lakes region, likely due to thinning and more variable snowpacks. However, a study by Dyer and Mote [62] suggested that minimal changes in North American snow depth has occurred in the November through January period from 1960–2000, but noted an earlier onset and acceleration of spring snowmelt in the March and April timeframe. While the long-term observational data presented in Hu and Feng [54] do not corroborate a reported trend of decreasing soil temperatures across Wisconsin and Michigan by Isard et al. [68], there were no observation stations across Wisconsin and Michigan included in the aforementioned analysis.

In our study, depending on the initial year for the calculation of linear trends (e.g., Fig. 11b compared to Fig. 11c), the magnitude of 10 cm soil temperature trends varied significantly across the Midwest. As with any type of linear trend analysis over time, the time period of choice can have a significant influence on the results. Agro-IBIS results for the 1948–2007 time period (Fig. 11b) illustrated a reduced warming signal, or no change in extreme minimum soil temperatures, across eastern Wisconsin and central lower Michigan, but are not necessarily indicative of a widespread cooling trend as suggested by Isard et al. [68]. We also analyzed Agro-IBIS extreme minimum soil temperature trends for the 50–60 cm soil depth, and did not find a significant difference from trends occurring at 10 cm (Fig. 11b). However, Agro-IBIS depicted several large areas of Missouri, Illinois, Indiana, and Ohio that exhibiting a cooling trend of annual average 10 cm soil temperatures (Fig. 11a), as well as the extreme minimum values (Fig. 11b) over a longer timeframe. Based on Agro-IBIS and soil temperature observation trends that agree, on average, for the 1981–2007 period for 36 station locations (SI, Table S1), we conclude that the coldest wintertime soil temperatures at 10 cm have been warming at a rate of approximately 0.8°C (10 yr)^−1^ to 0.9°C (10 yr)^−1^ over the last several decades. There is evidence to suggest that significant warming of soils and a reduction in soil frost would continue in the future, based on the modeling study of Sinha et al. [70]. They used future climate scenarios for the mid (2040–2069) and late (2070–2099) 21^st^ century to drive a macroscale land surface model and found that increased wintertime soil temperatures, increased frequency of freeze-thaw cycles, and a reduction in soil frost days across the region would occur with continued climate change.

### Challenges for ecosystem models

The model showed an overall good ability to simulate the dynamics of 10 cm soil temperatures observed at a number of locations in the Midwest (Figures 3–4), supporting the accuracy of our model estimates. The differences between observed soil temperatures in the tilled maize and switchgrass plots at the Arlington, WI site were attributed to a lack of an established residue layer in switchgrass experimental plots attributed to harvest of the majority of aboveground vegetation for three straight years. The simulation of interannual variability of annual extreme minimum temperatures at 10 cm proved more difficult than reproducing the average annual extreme soil temperatures at 10 cm. When we compared the frequency of occurrence of −3.5°C and −6.0°C soil temperatures with observations across the region (Figs. 5b,c), the model fit was not as good as we found when comparing the average annual extreme minimum temperatures (Fig. 5a). This result may be attributed to difficulties in simulating snow cover dynamics and the ability to capture the physical properties (i.e. density, compaction, water content) of snowpack on any particular day. Many ecosystem models currently do not have complicated dynamics such as the ability of standing vegetation or crop stubble to preferentially capture snow and lead to a deeper and longer duration of snow depth, which could lead to an accentuated insulating effect, higher soil temperatures, and decreased frost penetration [51], [71] and alter the surface albedo [72]. However, this is an area of great potential for future model improvement if more data are collected in a variety of land management settings. We also understand that physical processes in soils such as freeze-thaw cycles, and soil ice and frost formation can influence soil structure and infiltration [52], [53], [73], which ultimately affect heat transfer, and make soil temperature prediction particularly challenging at short timescales. Incorporation of these factors or refinement of current modeling approaches as more data become available should also improve ecosystem model realism.

While simulated snow depth agreed quite well with observations from November through March for the POTVEG simulation in the northern portion of the study region (Figs. 1a, 2a), snowmelt occurred more rapidly in March compared to observations when the model was parameterized with maize (row crops) or miscanthus (grasses). Additionally, the seasonal timing of the maximum snow depth occurred one month earlier than observations in those scenarios. Across the southern region (Fig. 1b, 2b), simulated snow depth agreed quite well with observations across all months and years for maize as prescribed vegetation, whereas the POTVEG yielded the poorest comparison with observations.

The connection between snowpack, the timing of snowmelt, and simulated soil temperatures (Fig. 3) was obvious. When comparing simulated soil temperatures to observations, the scenarios that yielded lower than observed snow depth and an earlier occurrence of snowmelt in March-April produced soil temperatures that were 5–10°C warmer than observed values during the late winter and spring (Fig. 3a). Thus, we conclude that accurate simulation of snowpack and snowmelt in ecosystem models is a crucial, but potentially overlooked, step towards simulating an accurate portrayal of changing energy balance at the soil surface when transitioning from winter to spring. This transition period typically coincides with rapid changes in ecosystem processes (e.g., net ecosystem exchange, ET), and the timing of warming and drying of soils impacts farmer management across the Corn Belt [74]. Subsequently, land cover and management choices further influence plant phenology, leaf area index, and canopy architecture, which all play important roles on changing radiation interception and energy balance [21], [38], [52], [61].

However, not all of the simulated error should probably be attributed to the model. When taking a closer examination of where and when the model performed well, we note that in the northern regions simulations of snow depth compared best with observations for the POTVEG simulations (Fig. 1a, 2a). Across the south, the opposite was true; the best comparisons with observations were found with simulations of maize (Fig. 1b, 2b). This is potentially a result of snow depth observations across the northern study having a higher likelihood of being collected in land cover/land use settings that are more reflective of natural vegetation (trees, shrubs, grasses), and therefore the snow depth in POTVEG simulations would likely agree better in those areas given closer agreement in plant phenology and LAI. Across the south, a higher proportion of land cover is in crops and might be the more likely land cover type where observations are collected, and therefore it might not be surprising that snow depth from the crop simulations across the southern region (Fig. 1b, 2b) compares the best across the south. Furthermore, accurate measurements of snow depth are known to be difficult to attain, attributed to a variety of factors, but recent improvements in technology may improve accuracy [75].

As discussed, there are several factors, concerning both modeling and observations, that contribute to perceived simulation error, and could call into question the statistical significance of the results. While using observational data across a wide range of research sites in the Midwest to validate the model would be considered a positive attribute of this study, we do not have a clear picture of the land-use history at those sites that could influence soil temperatures and we are focusing on the ability of the model to simulate the coldest (extreme) soil temperatures each year, which may not be sustained for more than a few days. We also know how difficult it is to simulate snow depth and density at any given site without having hourly weather data, and how influential hourly air temperature and other atmospheric conditions are in determining the ratio of liquid precipitation to accumulated snow depth. In Agro-IBIS, an air temperature threshold of 1.1°C is used to determine whether precipitation falls in liquid (rain) or frozen (snow) form, and a constant snow density is assumed. While the gridded climate data that we employed in this study are of extremely high quality and 5 min spatial resolution, some of the model bias for extremely cold soil temperatures (<−6.0°C) is likely induced by uncertainty in simulation of snowfall, snowmelt, and snow density, which is attributed to differences between the gridded climate data used to drive the model and what actually occurred at each site. For example, when additional model validation was performed at two specific locations (experimental stations in Wisconsin and Illinois) and implemented site-specific meteorological (hourly) and management data as drivers, the model agreed with observational data quite well, especially at the monthly time scale. Therefore, when the model had the best available land management and meteorological data to drive simulations, the comparisons were very strong.

The statistical significance of the results should be perceived as strong across large spatial regions like the Midwest US, and a good representation of how varied residue management impacts the typical average minimum soil temperatures. However, subtle changes in land management that effect soil structure, surface roughness, and the ability of the landscape to preferentially trap snow, as well as the short timescale temporal weather patterns, particularly those producing rapid changes in air temperature without snowpack, can lead to widely varying results. These temperature responses may be even greater than the magnitude of changes associated with varied residue management. The results presented here are robust as a broad generalization of what could happen at any particular Midwest US location in the context of residue management on soil temperatures based on the mean climate, but there are a range of other factors in any single winter season that could lead to a significant departure from the simulated mean responses.

### Conclusions

There are numerous factors that will influence the ability of miscanthus to overwinter in the first year or two after establishment, including rhizome size, end of season harvest management, planting depth, soil water content, rhizome moisture content at the end of the season, and soil characteristics [4], [7], [10]. However, there is no denying that residue management plays a significant role in soil thermal dynamics, particularly on wintertime soil temperatures, and therefore on miscanthus rhizome survival during the early stages of establishment. The results presented here illustrated that very small differences in thatch thickness, on the order of a few centimeters, lead to significant differences in minimum winter soil temperatures. Continued field research across a wide climate gradient, and assessing varied management scenarios, will help to fill the gaps in our understanding of rhizome winter survival. A potential wild card might be how long-term climate change impacts snowpack variability and annual minimum soil temperatures, the latter of which have been shown to be warming over the last several decades. In the case of miscanthus, producers could be presented with a new cropping system to support the production of renewable fuels in the future. However, they may be faced with dilemmas on how to best manage miscanthus in its establishment phase to ensure long-term survival while simultaneously remaining profitable.

## Supporting Information

Table S1 **Soil temperature observation sites used in model validation and minimum soil temperature assessment.** List of observation sites that were used to validate Agro-IBIS and for further assessment of annual minimum extreme 10 cm soil temperatures across the Midwest US. Soil surface refers to ground cover present; initial year is the beginning of the observation record used (starting January 1) and end year denotes the last year of the observation record used in this study (last day of record is December 31). Italicized and bolded station names denote the 36 locations that were used in an assessment of soil temperature trends simulated by Agro-IBIS.
(PDF)
