# Supplementary material for: Miscanthus Establishment and Overwintering in the Midwest USA: A Regional Modeling Study of Crop Residue Management on Critical Minimum Soil Temperatures
Source: PLoS One. 2013 Jul 3;8(7):e68847. doi: 10.1371/journal.pone.0068847 (PMC3700968; doi:10.1371/journal.pone.0068847)
Supplement: Table S1 — Soil temperature observation sites used in model validation and minimum soil temperature assessment. List of observation sites that were used to validate Agro-IBIS and for further assessment of annual minimum extreme 10 cm soil temperatures across the Midwest US. Soil surface refers to ground cover present; initial year is the beginning of the observation record used (starting January 1) and end year denotes the last year of the observation record used in this study (last day of record is December 31). Italicized and bolded station names denote the 36 locations that were used in an assessment of soil temperature trends simulated by Agro-IBIS. (PDF) [file pone.0068847.s001.pdf]

Table S1.

Station Name	State	Network	Soil Surface	Latitude	Longitude	Initial year	End year	Years in record
Ames	IA	Iowa Environmental Mesonet (IEM) / Iowa Ag Climate Network	bare	42.02	-93.77	1987	2011	25
Castana	IA	IEM	bare	42.06	-95.88	1989	2011	23
Cedar Rapids	IA	IEM	bare	41.91	-91.61	1998	2011	14
Chariton	IA	IEM	bare	41.10	-93.42	1989	2011	23
Crawfordsville	IA	IEM	bare	41.20	-91.49	1990	2011	22
Gilbert	IA	IEM	bare	42.10	-93.58	1987	2011	25
Kanawha	IA	IEM	bare	42.92	-93.78	1998	2011	14
Lewis	IA	IEM	bare	41.31	-95.17	1998	2011	14
Nashua	IA	IEM	bare	42.94	-92.57	1989	2011	23
Sutherland	IA	IEM	bare	42.93	-95.54	1989	2011	23
Belleville	IL	Illinois State Water Survey (ISWS)	grass	38.52	-89.84	1990	2011	22
Bondville	IL	ISWS	grass	40.05	-88.37	1991	2011	21
Brownstown	IL	ISWS	grass	38.95	-88.96	1990	2011	22
Carbondale	IL	ISWS	grass	37.69	-89.24	1991	2011	21
Champaign	IL	ISWS	grass	40.08	-88.24	1991	2011	21
DeKalb	IL	ISWS	grass	41.84	-88.85	1989	2011	23
Dixon Springs	IL	ISWS	grass	37.44	-88.67	1991	2011	21
Fairfield	IL	ISWS	grass	38.38	-88.39	1992	2011	20
Freeport	IL	ISWS	grass	42.28	-89.67	1990	2011	22
Kilbourne	IL	ISWS	grass	40.16	-90.09	1989	2011	23
Monmouth	IL	ISWS	grass	40.93	-90.72	1990	2011	22
Olney	IL	ISWS	grass	38.74	-88.10	1990	2011	22
Peoria	IL	ISWS	grass	40.71	-89.51	1994	2011	18
Perry	IL	ISWS	grass	39.80	-90.82	1990	2011	22
Rend Lake	IL	ISWS	grass	38.14	-88.92	1991	2011	21
Springfield	IL	ISWS	grass	39.72	-89.61	1989	2011	23
St. Charles	IL	ISWS	grass	41.90	-88.36	1989	2011	23
Stelle	IL	ISWS	grass	40.95	-88.16	1989	2011	23
Agry Center for Research	IN	Indiana Purdue Automated Agricultural Weather Station Network (PAAWS)	bare	40.55	-86.92	2003	2011	9
Davis Purdue Ag Center	IN	PAAWS	bare	40.25	-85.15	2003	2011	9
Northeast Purdue Ag Center	IN	PAAWS	bare	41.10	-85.38	2003	2011	9
Southeast Purdue Ag Center	IN	PAAWS	bare	39.03	-85.52	2003	2011	9
Southwest Purdue Ag Center	IN	PAAWS	bare	38.73	-87.48	2003	2011	9
Throckmorton	IN	PAAWS	bare	40.30	-86.90	2003	2011	9
Wanatah 2 WNW	IN	PAAWS	bare	41.45	-86.93	2003	2011	9
Colby	*KS*	*High Plains Regional Climate Center (HPRCC) Automated Weather Data Network (AWDN)*	*bare*	*39.38*	*-101.07*	*1985*	*2011*	*27*
Garden City	*KS*	*HPRCC AWDN*	*bare*	*37.98*	*-100.82*	*1985*	*2011*	*27*
Hays	*KS*	*HPRCC AWDN*	*bare*	*38.87*	*-99.33*	*1985*	*2011*	*27*
Hesston	*KS*	*HPRCC AWDN*	*bare*	*38.13*	*-97.40*	*1985*	*2011*	*27*
Hutchinson	KS	HPRCC AWDN	bare	37.93	-98.03	1988	2011	24
Manhattan #1	*KS*	*HPRCC AWDN*	*bare*	*39.20*	*-96.58*	*1984*	*2011*	*28*
Ottawa	*KS*	*HPRCC AWDN*	*bare*	*38.62*	*-95.28*	*1985*	*2011*	*27*
Parsons	*KS*	*HPRCC AWDN*	*bare*	*37.37*	*-95.28*	*1985*	*2011*	*27*
Rossville	KS	HPRCC AWDN	bare	39.12	-95.92	1988	2011	24
Scandia	*KS*	*HPRCC AWDN*	*bare*	*39.78*	*-97.78*	*1985*	*2011*	*27*
Silver Lake	*KS*	*HPRCC AWDN*	*bare*	*39.03*	*-95.70*	*1985*	*2011*	*27*
St. John	*KS*	*HPRCC AWDN*	*bare*	*37.93*	*-98.77*	*1985*	*2011*	*27*
Tribune	*KS*	*HPRCC AWDN*	*bare*	*38.47*	*-101.77*	*1985*	*2011*	*27*
Eldred	MN	HPRCC AWDN	bare	47.68	-96.82	1995	2011	17
Humboldt	MN	HPRCC AWDN	bare	48.88	-97.15	1995	2011	17
Perley	MN	HPRCC AWDN	bare	47.18	-96.68	1995	2011	17
Stephen	MN	HPRCC AWDN	bare	48.48	-96.70	1994	2011	18
Warren	MN	HPRCC AWDN	bare	48.13	-96.83	1995	2011	17
Rockport	MO	HPRCC AWDN	bare	40.47	-95.48	1991	2011	21
St. Joe	MO	HPRCC AWDN	bare	39.77	-94.92	1992	2011	20
Baker	ND	HPRCC AWDN	bare	48.17	-99.65	1993	2011	19
Beach	ND	HPRCC AWDN	bare	46.78	-103.97	1993	2011	19
Bottineau	ND	HPRCC AWDN	bare	48.82	-100.76	1994	2011	18
Bowman	ND	HPRCC AWDN	bare	46.20	-103.47	1993	2011	19
Cavalier	ND	HPRCC AWDN	bare	48.77	-97.75	1993	2011	19
Dazey	ND	HPRCC AWDN	bare	47.18	-98.13	1993	2011	19
Edgeley	ND	HPRCC AWDN	bare	46.32	-98.77	1993	2011	19
Forest River	ND	HPRCC AWDN	bare	48.30	-97.60	1991	2011	21
Hazen	ND	HPRCC AWDN	bare	47.30	-101.68	1993	2011	19
Hillsboro	ND	HPRCC AWDN	bare	47.35	-96.92	1993	2011	19
Langdon	ND	HPRCC AWDN	bare	48.77	-98.35	1988	2010	23
Linton	ND	HPRCC AWDN	bare	46.55	-100.47	1993	2011	19
Mohall	ND	HPRCC AWDN	bare	48.77	-101.53	1993	2011	19
Oakes	ND	HPRCC AWDN	bare	46.07	-98.10	1990	2011	22
Prosper	ND	HPRCC AWDN	bare	47.00	-97.12	1990	2011	22
Robinson	ND	HPRCC AWDN	bare	47.17	-99.80	1993	2011	19
Streeter	ND	HPRCC AWDN	bare	46.72	-99.45	1988	2011	24
Turtle Lake	ND	HPRCC AWDN	bare	47.57	-100.90	1993	2011	19
Wyndmere	ND	HPRCC AWDN	bare	46.27	-97.10	1990	2011	22
Ainsworth	*NE*	*HPRCC AWDN*	*bare*	*42.55*	*-99.82*	*1984*	*2011*	*28*
Alliance North	NE	HPRCC AWDN	bare	42.18	-102.92	1988	2011	24
Alliance West	NE	HPRCC AWDN	bare	42.02	-103.13	1988	2011	24
Arapahoe Prairie	NE	HPRCC AWDN	bare	41.48	-101.85	1987	2011	25
Arthur	*NE*	*HPRCC AWDN*	*bare*	*41.65*	*-101.52*	*1982*	*2011*	*30*
Beatrice	NE	HPRCC AWDN	bare	40.30	-96.93	1990	2011	22
Champion	*NE*	*HPRCC AWDN*	*bare*	*40.40*	*-101.72*	*1982*	*2011*	*30*
Clay Center (SC)	*NE*	*HPRCC AWDN*	*bare*	*40.57*	*-98.13*	*1982*	*2011*	*30*
Concord (NE)	*NE*	*HPRCC AWDN*	*bare*	*42.38*	*-96.95*	*1982*	*2011*	*30*
Curtisunsta	NE	HPRCC AWDN	bare	40.63	-100.50	1986	2011	26
Dickens	*NE*	*HPRCC AWDN*	*bare*	*40.95*	*-100.98*	*1982*	*2011*	*30*
Elgin	NE	HPRCC AWDN	bare	41.93	-98.18	1988	2011	24
Gordon	*NE*	*HPRCC AWDN*	*bare*	*42.73*	*-102.17*	*1984*	*2011*	*28*
Gudmundsen Rsrch	*NE*	*HPRCC AWDN*	*bare*	*42.07*	*-101.43*	*1982*	*2011*	*30*
Halsey	NE	HPRCC AWDN	bare	41.90	-100.15	1990	2011	22
Havelock	*NE*	*HPRCC AWDN*	*bare*	*40.85*	*-96.60*	*1983*	*2011*	*29*
Holdrege	NE	HPRCC AWDN	bare	40.33	-99.37	1988	2011	24
Lexingon	NE	HPRCC AWDN	bare	40.77	-99.73	1986	2011	26
Lincolnianar	NE	HPRCC AWDN	bare	40.83	-96.65	1986	2011	26
McCook	*NE*	*HPRCC AWDN*	*bare*	*40.23*	*-100.58*	*1982*	*2011*	*30*
Mead	*NE*	*HPRCC AWDN*	*bare*	*41.15*	*-96.48*	*1982*	*2011*	*30*
Mead Turf Farm	NE	HPRCC AWDN	bare	41.17	-96.47	1986	2011	26
North Platte	*NE*	*HPRCC AWDN*	*bare*	*41.08*	*-100.77*	*1982*	*2011*	*30*
ONeill	*NE*	*HPRCC AWDN*	*bare*	*42.47*	*-98.75*	*1985*	*2011*	*27*
Ord	*NE*	*HPRCC AWDN*	*bare*	*41.62*	*-98.93*	*1983*	*2011*	*29*

Scotts Bluff	NE	HPRCC AWDN	bare	41.88	-103.67	1991	2011	21
Sidney	*NE*	*HPRCC AWDN*	*bare*	*41.22*	*-103.02*	*1982*	*2011*	*30*
West Point	*NE*	*HPRCC AWDN*	*bare*	*41.85*	*-96.73*	*1982*	*2011*	*30*
Ashtabula	OH	Ohio Agricultural Research and Development Center (OARDC) Weather System	bare	41.88	-80.70	1986	2011	26
Columbus	OH	OARDC Weather System	bare	39.96	-83.00	1986	2011	26
Delaware	*OH*	*OARDC Weather System*	*bare*	*40.30*	*-83.07*	*1982*	*2011*	*30*
Jackson	*OH*	*OARDC Weather System*	*bare*	*39.05*	*-82.64*	*1982*	*2011*	*30*
Miami	OH	OARDC Weather System	bare	39.51	-84.73	1982	2006	25
Northwest	*OH*	*OARDC Weather System*	*bare*	*41.29*	*-83.84*	*1982*	*2011*	*30*
Piketon	OH	OARDC Weather System	bare	39.07	-83.01	1993	2011	19
Western	*OH*	*OARDC Weather System*	*bare*	*39.86*	*-83.67*	*1982*	*2011*	*30*
Wooster	*OH*	*OARDC Weather System*	*bare*	*40.78*	*-81.93*	*1982*	*2011*	*30*
Beresford	SD	HPRCC AWDN	bare	43.07	-96.93	1988	2011	24
Britton	SD	HPRCC AWDN	bare	45.80	-97.72	1998	2011	14
Brookings	*SD*	*HPRCC AWDN*	*bare*	*44.32*	*-96.00*	*1983*	*2011*	*29*
Cottonwood	SD	HPRCC AWDN	bare	43.97	-101.87	1988	2011	24
Gettysburg	*SD*	*HPRCC AWDN*	*bare*	*45.02*	*-99.97*	*1983*	*2011*	*29*
Nisland	SD	HPRCC AWDN	bare	44.68	-103.57	1988	2011	24
Oacoma	*SD*	*HPRCC AWDN*	*bare*	*43.75*	*-99.55*	*1984*	*2011*	*28*
Pierre	SD	HPRCC AWDN	bare	44.28	-100.00	1991	2011	21
Redfield	*SD*	*HPRCC AWDN*	*bare*	*44.87*	*-98.52*	*1983*	*2011*	*29*
South Shore	SD	HPRCC AWDN	bare	45.10	-96.92	1988	2011	24
Arlington	WI	Wisconsin (WI) Automated Weather Observation Network (AWON)	mixed	43.31	-89.38	1988	2011	24
Chetek	WI	WI AWON	mixed	45.30	-91.53	1988	1998	11
Hancock	WI	WI AWON	mixed	44.12	-89.53	1989	2011	23
Spring Green	WI	WI AWON	mixed	43.18	-89.61	1990	2011	22
